# New approaches to screening and management of neonatal hypoglycemia based on improved understanding of the molecular mechanism of hypoglycemia

**DOI:** 10.3389/fped.2023.1071206

**Published:** 2023-03-10

**Authors:** Charles A. Stanley, Paul S. Thornton, Diva D. De Leon

**Affiliations:** ^1^ Congenital Hyperinsulinism Center and Division of Endocrinology and Diabetes, Children’s Hospital of Philadelphia, Philadelphia, PA, United States; ^2^ Department of Pediatrics, Perelman School of Medicine at the University of Pennsylvania, Philadelphia, PA, United States; ^3^ Congenital Hyperinsulinism Center, Division of Endocrinology, Cook Children’s Medical Center, Fort Worth, TX, United States; ^4^ Department of Pediatrics, Texas Christian University Burnett School of Medicine, Fort Worth, TX, United States

**Keywords:** glucose, insulin, ketones, newborns, brain damage

## Abstract

For the past 70 years, controversy about hypoglycemia in newborn infants has focused on a numerical “definition of neonatal hypoglycemia”, without regard to its mechanism. This ignores the purpose of screening newborns for hypoglycemia, which is to identify those with pathological forms of hypoglycemia and to prevent hypoglycemic brain injury. Recent clinical and basic research indicates that the three major forms of neonatal hypoglycemia are caused by hyperinsulinism (recognizing also that other rare hormonal or metabolic conditions may also present during this time frame). These include transitional hypoglycemia, which affects all normal newborns in the first few days after birth; perinatal stress-induced hypoglycemia in high-risk newborns, which afflicts ∼1 in 1,200 newborns; and genetic forms of congenital hyperinsulinism which afflict ∼1 in 10,000–40,000 newborns. (1) Transitional hyperinsulinism in normal newborns reflects persistence of the low glucose threshold for insulin secretion during fetal life into the first few postnatal days. Recent data indicate that the underlying mechanism is decreased trafficking of ATP-sensitive potassium channels to the beta-cell plasma membrane, likely a result of the hypoxemic state of fetal life. (2) Perinatal stress-induced hyperinsulinism in high-risk infants appears to reflect an exaggeration of this normal low fetal glucose threshold for insulin release due to more severe and prolonged exposure to perinatal hypoxemia. (3) Genetic hyperinsulinism, in contrast, reflects permanent genetic defects in various steps controlling beta-cell insulin release, such as inactivating mutations of the *K*_ATP_-channel genes. The purpose of this report is to review our current knowledge of these three major forms of neonatal hyperinsulinism as a foundation for the diagnosis and management of hypoglycemia in newborn infants. This includes selection of appropriate interventions based on underlying disease mechanism; combined monitoring of both plasma glucose and ketone levels to improve screening for infants with persistent forms of hypoglycemia; and ultimately to ensure that infants at risk of persistent hyperinsulinemic hypoglycemia are recognized prior to discharge from the nursery.

## 1. Introduction

There are three fundamental flaws in the current approach to neonatal hypoglycemia which severely limit its utility: The first flaw is the underlying presumption that infants and children have lower brain requirements for glucose compared to adults. However, the rates of glucose utilization, determined primarily by brain consumption, are similar per kilogram brain weight in newborn infants and adults (1).

**Figure 1:**
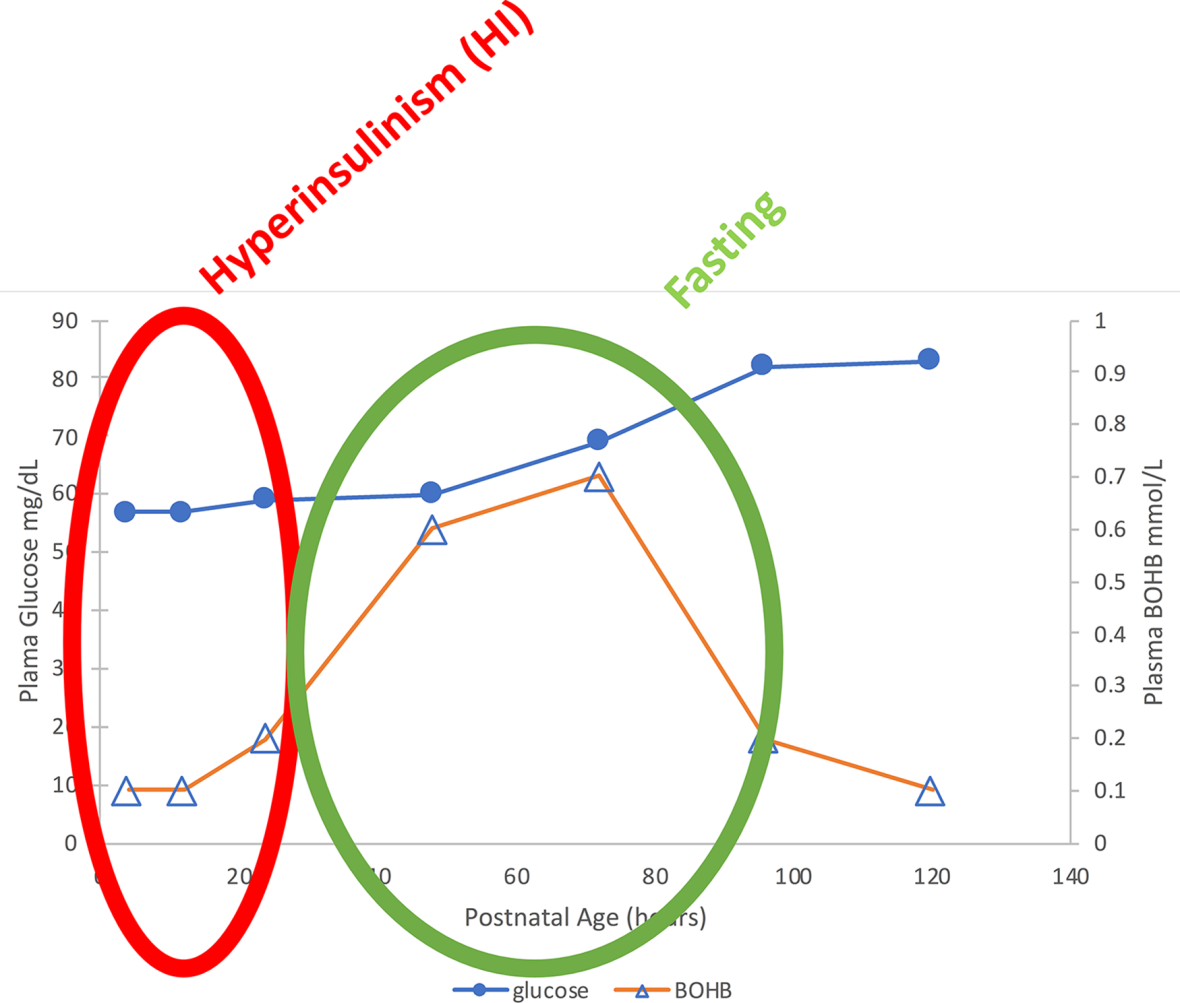
Median plasma glucose and BOHB in normal breastfed newborns. Note the two phases of “neonatal hypoglycemia”: (1) Birth—24 h: Hypoglycemia with hypo-ketonemia due to transient Hyperinsulinism; (2) 36–72 h in breastfed babies: Milder hypoglycemia with hyper-ketonemia = “Fasting” (4).

The second flaw in the current approach to neonatal hypoglycemia is the assumption that neonatal hypoglycemia can be “defined” as a single glucose value. This is an outmoded notion, as pointed out in the recent hypoglycemia guidelines of the Endocrine Society and Pediatric Endocrine Society (2, 3).The current so-called “definition” of neonatal hypoglycemia, for instance, does not take into account the fact that normal breastfed neonates have hypo-ketonemic hypoglycemia during the first 24–36 h, but then may have hyper-ketonemic hypoglycemia between 36 and 72 h until taking sufficient breast milk (see Figure 1) (4). In addition, the current “definition” of neonatal hypoglycemia is admittedly not based on scientific grounds, but instead is claimed to be an “operational definition” aimed at avoiding unnecessary interventions (5). Efforts have been made to validate a single glucose value for defining neonatal hypoglycemia by neurodevelopmental outcome studies of varying durations; however, these are merely association studies that do not relate outcomes to either specific diagnosis for the etiology of hypoglycemia, specific hypoglycemic events, or level of plasma glucose and availability of alternative brain fuels (ketones or lactate) and have yielded conflicting data on any specific glucose value (6–9).

The third and most important flaw in the current approach to neonatal hypoglycemia, based on the so-called “operational” definition of neonatal hypoglycemia, is that it has failed to serve its most important purpose: early detection and rapid effective treatment to prevent hypoglycemic brain injury in babies with severe and persistent disorders of hypoglycemia. The frequency of long-term developmental delay due to failure to diagnose the etiology of hypoglycemia and to adequately treat hypoglycemia in the most common of these disorders, congenital hyperinsulinism, has remained at 30%–50% for the last 50 years or more, despite major improvements in technologies for glucose testing and treatment (10–12).

The purpose of this report is to encourage the development of new approaches to diagnosis and management of the various forms of neonatal hypoglycemia based on the physiologic and biochemical mechanism(s) of hypoglycemia in newborn infants. As we show below, the three most common forms of hypoglycemia in newborn infants are all caused by altered regulation of pancreatic beta-cell insulin secretion: (1) transitional hypoglycemia in normal newborns (hereafter termed Transitional HI), (2) prolonged neonatal hyperinsulinism in high-risk neonates (hereafter termed Perinatal Stress-Induced HI), and (3) congenital genetic forms of hyperinsulinism (hereafter termed Genetic HI). This review will focus on utilizing the standard fasting systems approach for differentiating hyperinsulinism from other types of hypoglycemia based on disease mechanism; a description of the three forms of hyperinsulinism in newborn infants; new information on the molecular mechanism of hypoglycemia in normal and high-risk newborns; and conclude by suggesting potential approaches to improved screening and treatment for persistent hypoglycemia disorders to reduce the risk of permanent hypoglycemic brain injury in these babies. The authors recognize that other etiologies for hypoglycemia presenting in the neonatal period such as fatty acid oxidation disorders, hypopituitarism or the glycogen storages disorders must also be ruled out when evaluating neonatal hypoglycemia, however, this is not the focus of this article.

## 2. The three major forms of neonatal hypoglycemia are all due to hyperinsulinism

### 2.1. Transitional neonatal hyperinsulinism (transitional HI)

Transitional HI in normal newborns is a hypoketotic form of hypoglycemia due to persistence of fetal insulin regulation by pancreatic beta-cells (13).

**Figure 2:**
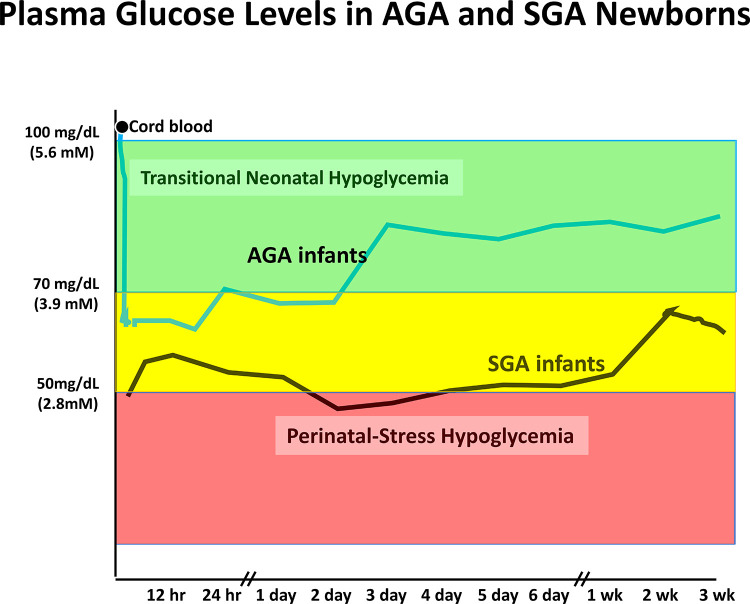
Neonatal hypoglycemia in normal AGA newborns and high-risk SGA infants. Mean plasma glucose prior to birth is similar to maternal level and drops to range of 55–70 mg/dl during first 12–18 h before rising into normal extra-uterine glucose range between 24 and 48 h. Mean glucose drops lower in high-risk neonates (SGA) and remains below normal range for several days up to a few weeks. Similar prolonged neonatal hypoglycemia occurs in other high-risk neonates due to birth asphyxia, maternal hypertension/toxemia, etc. Redrawn from (14).

**Figure 3:**
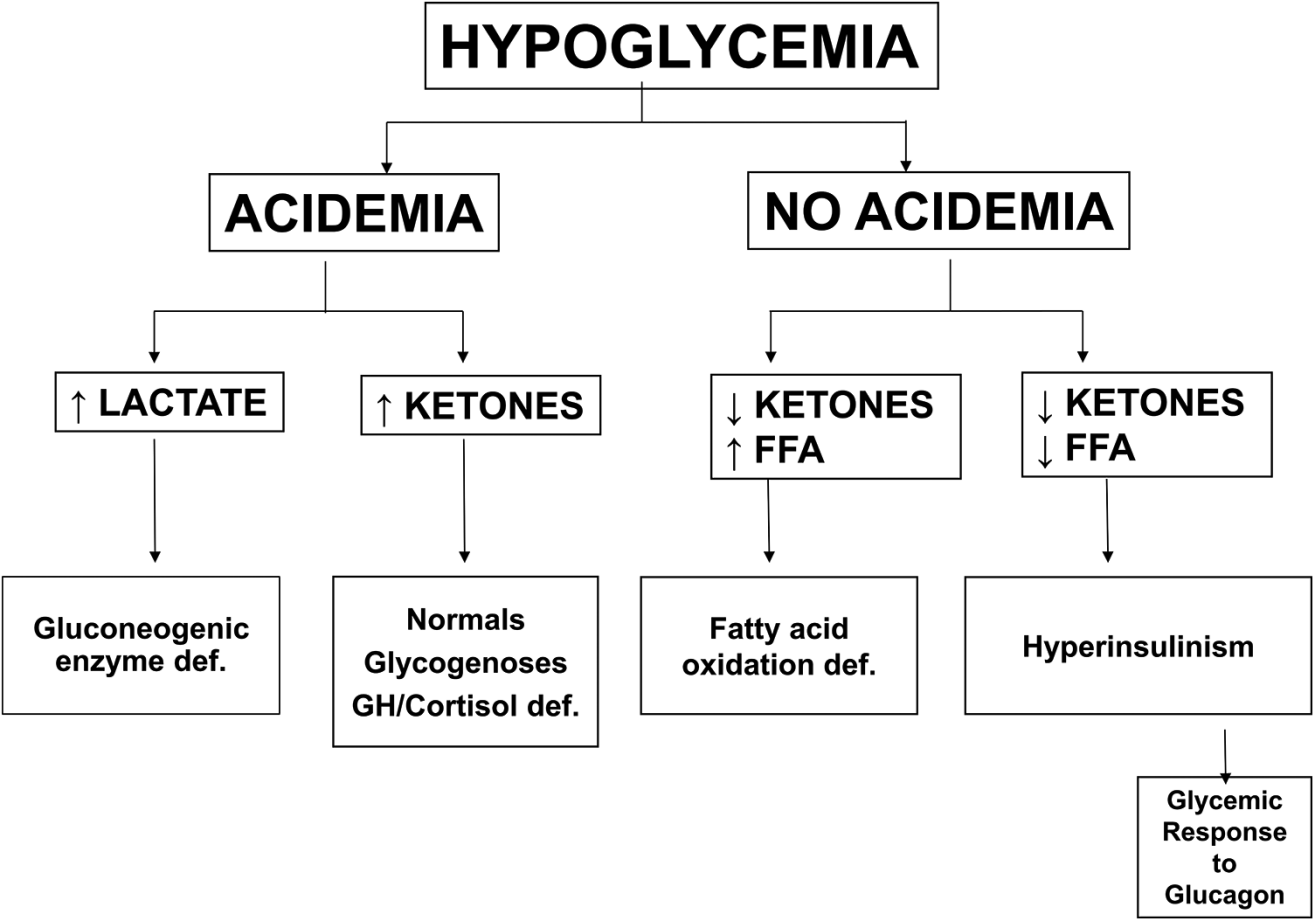
Differential diagnosis of hypoglycemia based on plasma metabolic fuel responses. Note that Hyperinsulinism is associated with suppression of ketones and FFA and retention of a large glycemic response to injection of glucagon. Abbreviations: FFA (free fatty acids), GH/Cortisol def (growth hormone and/or cortisol deficiency).

Plasma glucose levels in the fetus are maintained close to maternal levels up to the time of delivery by efficient facilitated transport of glucose across the placenta from the maternal to the fetal circulation. Immediately after delivery, plasma glucose in the newborn infant rapidly drops approximately 20 mg/dl (1.1 mmol/L) and remains low for the first 24–36 h of life (see Figure 2). This period of hypoglycemia in normal newborns has the characteristic features of hyperinsulinism: suppression of plasma levels of ketones and large glycemic responses to glucagon or epinephrine due to inhibition of glycogenolysis in the liver by insulin (See Figure 3 and PES guidelines) (13). This reflects persistence of a lower pancreatic beta-cell glucose threshold for insulin release during fetal life which serves to support the high rates of fetal growth. For this reason, it can be appropriately termed, Transitional HI.

Recent data from Harris and colleagues in New Zealand on post-natal plasma glucose and ketone levels (4) (Figure 1) demonstrate that in breast-fed newborns, there are actually two phases of neonatal hypoglycemia. The first phase is the period of hypo-ketonemic hypoglycemia due to Transitional HI during the first 24–36 h after birth. In breast-fed newborns, this is followed by a second phase of hyper-ketonemic hypoglycemia between 48 and 72 h after birth which then resolves as plasma glucose rises into the normal range for older infants and children. In contrast to the first phase of Transitional HI, the second phase reflects a normal fasting adaptation to the limited supply of breast milk before 3–5 days of age; thus, this phase may be absent in bottle-fed infants receiving greater intake in the first days after delivery. Figure 2 indicates that the period of Transitional HI in normal newborns is fully resolved by 36–48 h of age; as discussed later, this may be an appropriate age for detecting persistent hyperinsulinism disorders before discharge to home.

The clinical features of Transitional HI in normal newborns indicate that the underlying mechanism is a lower glucose threshold for beta-cell insulin release during fetal life (13). The consequences of a lower beta-cell glucose threshold are illustrated by the hyperinsulinism disorder caused by gain-of-function mutations in glucokinase (GCK-HI), the beta-cell glucose sensor (see below). Children with GCK-HI have low, but quite stable baseline plasma glucose levels in the range of 50–65 mg/dl (2.8–3.6 mmol/L); this is close to the thresholds for symptoms of hypoglycemia. Similarly, in Transitional HI, plasma glucose levels tend to be quite stable and are relatively unaffected by feeding (13). Lubchenko and Bard found that of all the babies in a nursery the median glucose was 50–60 mg/dl (2.8–3.3 mmol/L) and that 11% babies had serum glucose below 30 mg/dl (1.7 mmol/L) at 8 h of age (15) and that by 72 h of age <0.05% babies had glucose levels <50 mg/dl (<2.8 mmol/L). They concluded that a serum glucose level of <30 mg/dl (<1.7 mmol/L) served to identify sick infants since this level was primarily associated with intrauterine growth retardation (IUGR) and perinatal stress. As described below, recent studies of the molecular mechanism of Transitional HI show that the lower glucose threshold for insulin release reflects decreased trafficking of the beta-cell K_ATP_ channel to the plasma membrane surface, probably as a consequence of the hypoxemic environment of fetal life (16).

### 2.2. Perinatal stress-induced hyperinsulinism in high-risk newborns (perinatal stress-induced HI)

This is the second most common form of neonatal hypoglycemia and affects approximately 1 in 1,000 newborns for variable lengths of time after birth (17). These infants show the typical features of hypoglycemia due to hyperinsulinism, including suppressed levels of plasma ketones and increased rates of glucose utilization, sometimes reaching as high as 20–30 mg/kg/min (18). It has been recognized since the 1960s that some groups of high-risk newborns have more profound and longer-lasting hypoglycemia after birth than normal infants. Cornblath described this in “low birthweight” (SGA) babies, whose period of postnatal hypoglycemia lasted for up to several weeks (19). Lubchenko and Bard also described a higher incidence of hypoglycemia in both term and preterm infants with IUGR and birth asphyxia (15) compared to non-stressed AGA infants. Severe hypoglycemia was also described in the early 1970s in babies with fetal anemia due to Rh-incompatibility (Erythroblastosis Fetalis) and shown to be due to hyperinsulinism (20). Leonard and colleagues reported severe hyperinsulinism lasting for several weeks after birth in babies with birth asphyxia and with intra-uterine growth retardation/SGA birthweight (21). Hoe et al. and Sigal et al. reported prolonged hyperinsulinism in large groups of babies with various types of perinatal stress, including SGA, birth asphyxia, and maternal hypertension (18, 22). In some of these babies with Perinatal Stress-Induced HI, hypoglycemia may be severe enough to require treatment with very high rates of glucose infusion and/or diazoxide (18). Hypoxemia seems to be the common feature in conditions associated with Perinatal Stress-Induced HI and studies described below of the effects of hypoxemia and the Hypoxia Inducible Factor (HIF) suggest that Perinatal Stress-Induced HI and Transitional HI may share the same molecular mechanism of beta-cell insulin dysregulation.

### 2.3. Genetic forms of hyperinsulinism (congenital HI)

**Figure 4:**
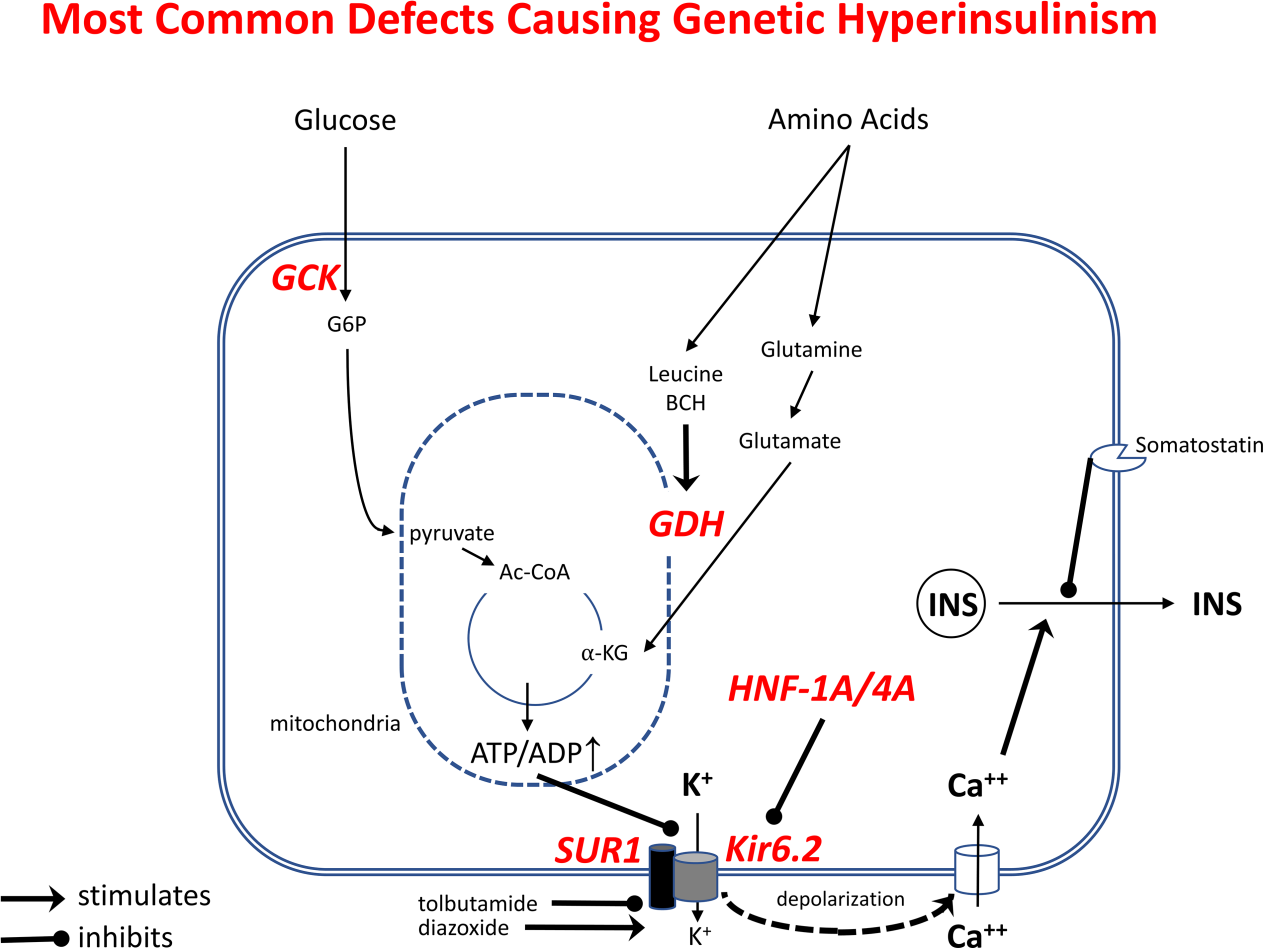
Most common genetic defects causing Genetic Hyperinsulinism. Outlined are the pathways stimulating beta-cell insulin release by glucose and amino acids. Oxidation of these fuels in mitochondria increases ATP/ADP ratio, leading to inhibition of K^+^ efflux *via* plasma membrane ATP-sensitive KATP-channel, membrane depolarization, Ca^++^ influx, and release of insulin from storage granules. Tolbutamide stimulates insulin release by closing K_ATP_-channels; diazoxide inhibits insulin release by opening K_ATP_-channels. Genetic Hyperinsulinism can be caused by inactivating mutations of K_ATP_-channel subunits (SUR1 or Kir6.2, encoded by *ABCC8* and *KCNJ11*) and by activating mutations of glucokinase (GCK) or glutamate dehydrogenase (GDH, encoded by *GLUD1*); inactivating mutations of HNF1A or HNF4A cause hyperinsulinism by decreasing gene expression of K_ATP_-channel subunits.

Congenital HI due to genetic defects in beta-cell insulin regulation is the most common form of persistent neonatal hypoglycemia. Although the incidence of Genetic HI is commonly quoted as 1:25,000 to 1:45,000 (23–26), it may be as high as 1 in 5,000–10,000, based on the population frequency of some dominant K_ATP_-channel mutations. A total of 39 dominant or recessive genetic loci have been associated with Genetic HI, including several syndromes with identifiable physical features, such as macroglossia and hemihypertrophy in Beckwith-Wiedemann syndrome. The most common hyperinsulinism genes are shown in Figure 4 which indicates their locations in the pathways controlling glucose and amino acid stimulated insulin secretion; this diagram also provides a background for defining the molecular mechanisms of hyperinsulinism in normal newborns and in high-risk neonates, described below. The most common genetic defects are in the *ABCC8* and *KCNJ11* genes that encode the two subunits of the K_ATP_ potassium channel (SUR1 and Kir6.2, respectively), which triggers the release of insulin during stimulation with metabolic fuels, such as glucose or amino acids (27). The second most commonly affected genes are *GCK*, the beta-cell glucose sensor (28), and *GLUD1*, encoding glutamate dehydrogenase (GDH), which mediates stimulation of insulin release by the amino acid leucine and its non-metabolizable analog, 2-aminobicyclo-(2,2,1)-heptane-2-carboxylic acid (BCH) (29). Dominant mutations of the *HNF4A* or *HNF1A* transcription factor genes cause HI, at least in part, due to decreased expression of the K_ATP_-channel; the hyperinsulinism usually resolves during infancy, but may evolve into diabetes in the second or third decade of life (MODY1 and MODY3), so affected infants frequently have a history of type 2 diabetes in a parent or other relative (30). Unlike the syndromic forms of hyperinsulinism, the common genetic forms of HI shown in Figure 4 have no abnormal clinical features, apart from large birthweight due to the growth-promoting effects of fetal hyperinsulinemia; these infants are therefore at extremely high risk of permanent hypoglycemic brain injury, because of delays in recognition and treatment.

## 3. Molecular mechanisms of transitional HI in normal newborns and perinatal stress-induced HI in high-risk neonates

### 3.1. Evidence that transitional HI is due to reduced beta-cell glucose threshold for insulin release secondary to decreased K_ATP_ channel trafficking

In order to understand the dysregulation of insulin secretion causing Transitional and Perinatal Stress-Induced HI it is helpful to understand the normal regulation of insulin secretion. Insulin secretion in the mature beta cell is tightly regulated by the K_ATP_ channel, which couples the metabolic state of the cell to membrane potential (and thus, insulin secretion) by sensing changes in ATP concentration. Glucose is transported into the beta cell by a glucose transporters (GLUT1 and GLUT2) and phosphorylated in a concentration dependent manner by glucokinase (GCK) to enter glycolysis and the Krebs cycle (the Citric Acid cycle) to produce ATP. The increase in ATP/ADP triggers closure of the K_ATP_ channel causing the beta cell membrane to depolarize allowing calcium to enter through voltage gated calcium channels. This then stimulates release of insulin into the circulation and lowers plasma glucose (Figure 4). As plasma glucose falls, there is less transport into the beta cell, less phosphorylation and less ATP generated. The K_ATP_ channel then opens and prevents depolarization of the cell membrane, preventing calcium transport into the cell and insulin secretion is turned off. Thus, lack of functional K_ATP_ channels results in constant depolarization and insulin secretion, uncoupling glucose concentration and insulin release (31).

**Figure 5:**
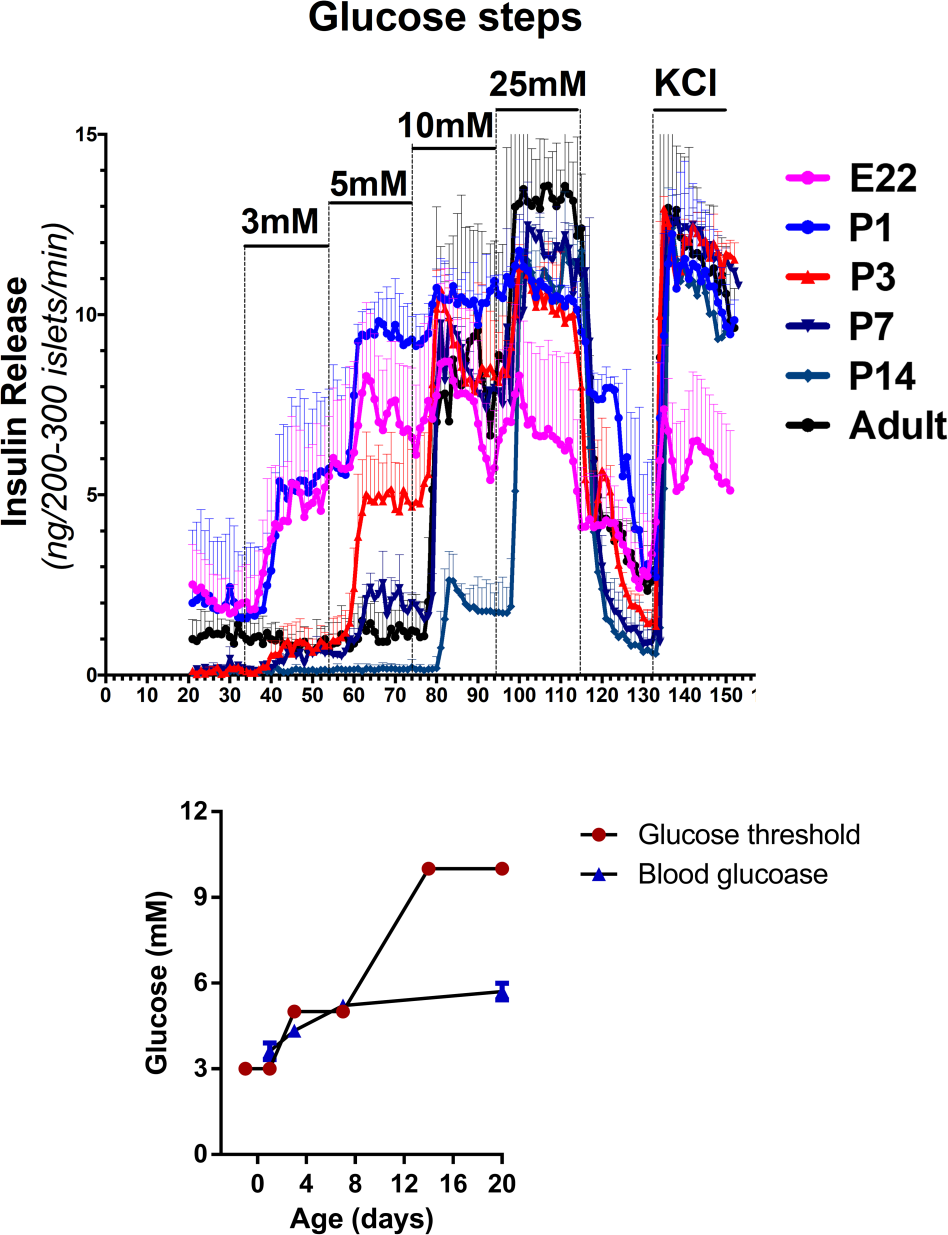
Developmental shift in glucose threshold for insulin release in fetal and early neonatal islets. Threshold for glucose stimulated insulin release is lower in fetal and neonatal islets compared to islets from older neonates and adults. Freshly isolated rat islets were perifused with a step-wise ramp stimulation by glucose from 3 mM to 25 mM, followed by maximal insulin release with KCl. Glucose thresholds were defined by the lowest glucose concentration stimulating insulin release greater than baseline glucose-free perifusion. The inset compares the time course of changes in mean plasma glucose concentration and mean glucose threshold for islet insulin release.

Studies in rodents have provided insights into the mechanisms regulating insulin secretion in the perinatal period. As shown in Figure 5, isolated islets from fetal rats at term (Embryonic Day 22, E22) release insulin at a glucose threshold level of 3 mmol/L, in contrast to adult islets which have a threshold for insulin release of 10 mmol/L. The glucose threshold for insulin release increases quickly over the first 1–3 days after birth and reaches adult levels by two weeks of age (P14). The inset in Figure 5 shows that in the rodent model, the time course of increase in beta-cell glucose threshold closely parallels the post-natal rise of plasma glucose levels. These results are consistent with many reports of a low glucose threshold for insulin secretion in fetal islets from humans, as well as rodents and other species, thus, suggesting that this phenomenon occurs across species (32, 33). They support the concept that Transitional HI in normal newborns is caused by persistence of the low glucose threshold for insulin release of fetal beta-cells.

**Figure 6:**
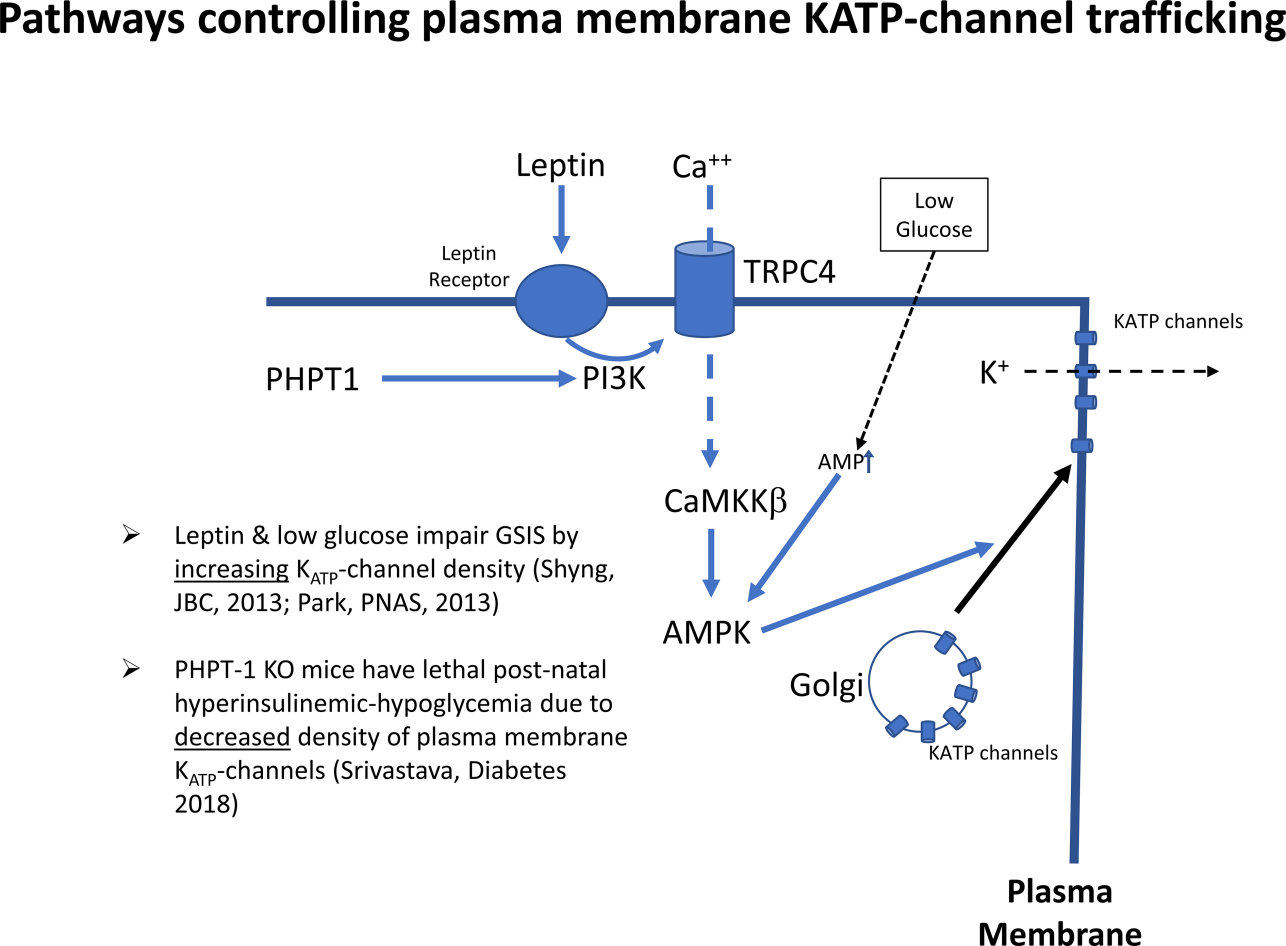
Signals controlling K_ATP_-channel trafficking from Golgi to plasma membrane. K_ATP_ channels traffic to the plasma membrane in response to activation of AMP-kinase by the calcium/calmodulin protein kinase 2, (CaMKKß). Abbreviations: PHPT1, protein histidine phosphatase 1; PI3K, phosphatidyl-inositol 3 kinase; TRPC4, transient receptor potential cation channel subfamily C member 4; AMPK, AMP-activated protein kinase; KATP, ATP-sensitive potassium channel.

Interestingly, insulin responses to tolbutamide stimulation, a K_ATP_ channel inhibitor, were markedly lower in P1 compared to P14 islets (not shown), thus indicating that the site of the lower glucose and amino acid thresholds for insulin release in this rodent model may be the K_ATP_-channel itself. We propose that the same mechanism is responsible for Transitional HI. Furthermore, patch-clamps of dispersed beta-cells from P3 rat pups show lower potassium ion currents compared to P14 beta-cells (not shown). This indicates that Transitional HI in normal newborns is caused by decreased numbers of K_ATP_-channels on the beta-cell plasma membrane surface. This is in accord with several studies showing that regulation of trafficking of K_ATP_-channels from the Golgi to the surface of the plasma membrane plays a major role in the responsiveness of beta-cells to glucose (Figure 6). Leptin and low glucose decrease beta-cell responses to glucose by increased trafficking of K_ATP_-channels from the Golgi to the plasma membrane *via* a pathway involving activation of AMPK. Conversely, disruption of this pathway by ablation of a plasma membrane phosphohistidine phosphatase, PHPT1, impedes Ca^++^ mediated activation of AMPK leading to decreased K_ATP_-channel trafficking and lethal neonatal hypoglycemia (34). Details on how this pathway may be involved in decreased K_ATP_-channel trafficking in Transitional HI deserves further investigation.

### 3.2. Evidence that hypoxia and the HIF pathway are signals controlling the mechanisms of both transitional HI and perinatal-stress associated HI

**Figure 7:**
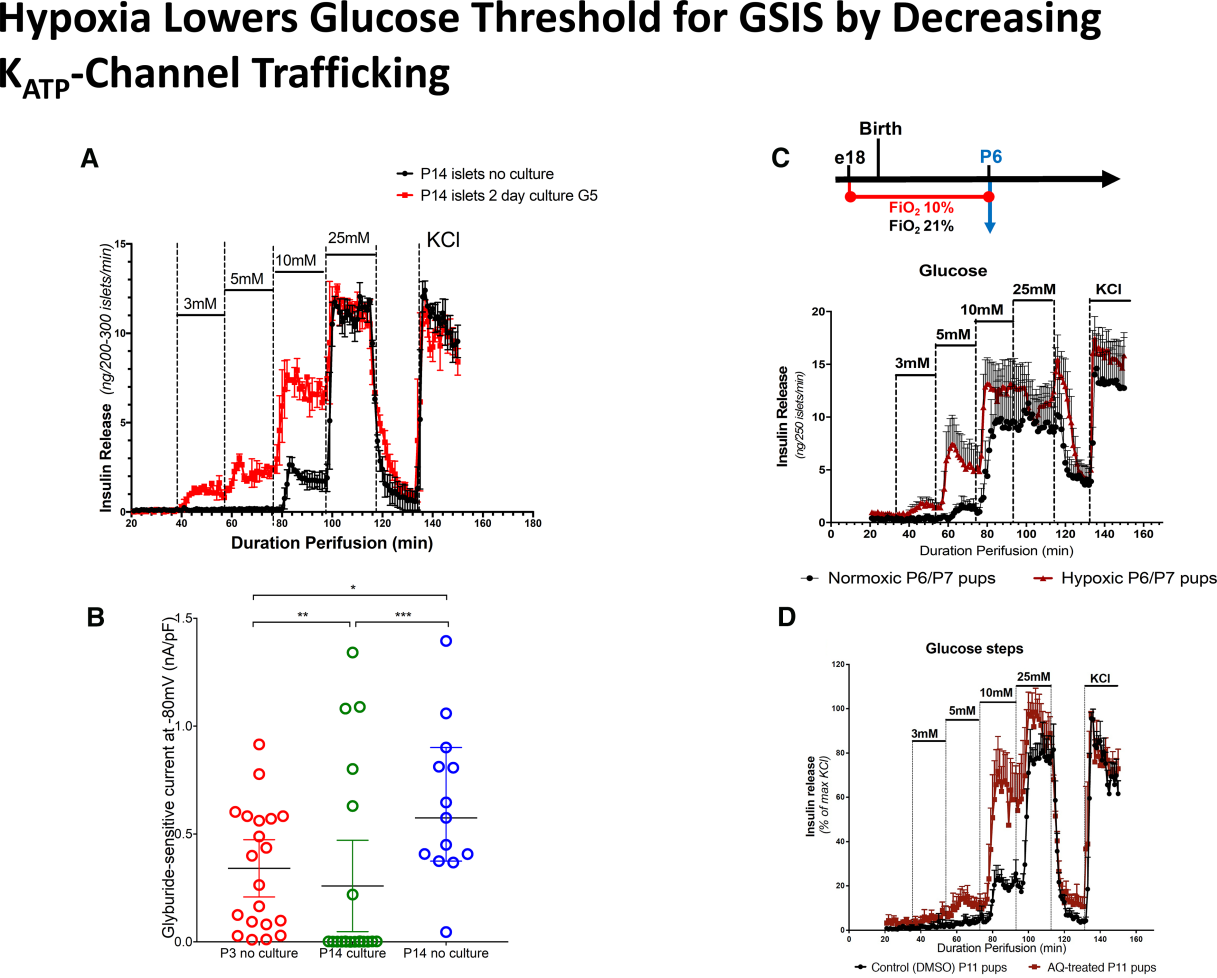
Hypoxia lowers glucose threshold for insulin release by decreasing K_ATP_-channel trafficking. (**A**) Hypoxia induced by culture of isolated islets for 2 days lowers the glucose threshold for glucose stimulated insulin secretion (GSIS). (**B**) Islet culture for 2 days reduces K_ATP_-channel trafficking compared to P14 islets without culture, similar to P3 islets without culture. (**C**). Exposure to hypoxia (FiO2 10%) from embryonic day E18 until postnatal day P6 reduces glucose threshold for GSIS. (**D**) Adaptaquin (AQ) treatment to mimick hypoxia by stabilizing Hypoxia Inducible Factor (HIF) lowers glucose threshold for insulin release in P11 islets.

There are good reasons to suspect that hypoxia and the Hypoxia Inducible Factor (HIF) pathways signal the low fetal glucose threshold for insulin release, since the fetus develops in a hypoxic environment which is relieved after the baby is born. This is supported by the following studies [available for viewing on-line (16)]. (1) As shown 20 years ago by Matschinsky and Davis (35) (Figure 7A, B), exposure of freshly isolated islets to the relatively hypoxic environment of tissue culture for 24–48 h results in a downward shift of the glucose threshold for insulin release from 7 mmol/L to 3 mmol/L (35, 36). This downward shift in glucose threshold is associated with a decrease in plasma membrane K_ATP_-channel density, suggesting a role for hypoxia as a signal for lowering the glucose threshold for insulin secretion. (2) Similarly, exposure of rodents to environmental hypoxia (10% O_2_) from embryonic day E18 to postnatal day P7 results in a downward shift of the islet glucose threshold from 10 mmol/L down to 3–5 mmol/L (Figure 7C) (37). (3) Direct activation of the HIF pathway by inhibiting prolyl-hydroxylase degradation of HIF with Adaptaquin from postnatal day P7 to P10 lowers the glucose threshold for insulin from 10 down to 5 mmol/L (Figure 7D). These results are consistent with the report from rodent studies that constitutive activation of the HIF pathway *in utero* by ablation of von Hippel-Lindau factor (vHL) to block proteolysis of HIF results in severe hypoglycemia (38).

An important role for the HIF-pathway as the signal for lower K_ATP_-channel trafficking and lower glucose threshold for insulin secretion is also indicated by the clinical observation that many of the conditions associated with perinatal-stress related hyperinsulinism involve hypoxia and/or reduced placental circulation (maternal toxemia, erythroblastosis fetalis, birth asphyxia, intrauterine growth retardation, etc) (18, 20). It is possible that hypoxia and the HIF pathway interact with the AMPK pathway controlling K_ATP_ channel trafficking shown in Figure 6. Further studies to examine these mechanisms may also identify targets for intervention that could be clinically relevant in babies with severe or prolonged forms of neonatal hyperinsulinism.

### Molecular mechanisms of transitional HI and perinatal stress-induced HI: summary

In brief, the above studies show that postnatal hypoglycemia associated with Transitional HI in normal newborns is caused by a lower beta-cell glucose threshold for insulin, which reflects decreased plasma membrane surface expression of ATP-sensitive K channels. The hypoxia/Hypoxia Inducible Factor (HIF) pathway appears to be an important signal for this decreased K_ATP_ trafficking in the fetus and early newborn period.

A clinical example of the effects of decreased beta-cell membrane surface expression of K_ATP_ channels is Genetic HI associated with dominant mutations of the HNF1A transcription factor that cause MODY 3. Islets from affected patients have been recently documented to have reduced expression of K_ATP_ channel subunits (39). Similarly, deletion of *HNF4A* (the MODY 1 gene) in a mouse model results in decreased expression of the Kir6.2 subunit of the K_ATP_ channels (40). The reduction in K_ATP_-channels in MODY1 patients results in a flattening of the insulin-glucose secretion curve (41) so that insulin release is not appropriately stimulated at high glucose levels (hence, glucose intolerance and diabetes, as insulin requirements increase in MODY1 patients later in life), but is also not appropriately suppressed at low glucose levels (hence, hyperinsulinemic-hypoglycemia in early infancy in patients with *HNF4A* mutations). Complete absence of plasma membrane K_ATP_ channels due to null mutations of the K_ATP_ channel subunits, results in more extreme flattening of glucose-insulin response curve with very severe neonatal hypoglycemia (as well as glucose intolerance later in life) (42).

An important implication of these findings is that Transitional HI in normal newborns and persistent hypoglycemia in high-risk newborns (Perinatal Stress-Induced HI) share the same mechanism; the latter may represent a more persistent suppression of beta-cell K_ATP_ channel trafficking. This implies that these two forms of neonatal hypoglycemia cannot be distinguished on the first day of life by biochemical testing, since they merely reflect extreme ends of the spectrum of persistent fetal hyperinsulinism and share the typical diagnostic features of hyperinsulinism (low glucose with suppressed ketone levels). As described below, it may be possible to develop improved methods for identifying babies with a persistent hypoglycemia disorder prior to discharge to home by monitoring the plasma levels of ketones, as well as glucose, during the first 2–3 days after birth.

## 4. Opportunities for improving management of transitional, perinatal stress-induced, and genetic HI

### 4.1. Improved selection of rational glucose thresholds for intervention and for treatment targets in neonatal hypoglycemia

One of the major deficiencies of current clinical practice guidelines for neonatal hypoglycemia is the use of a single, arbitrary glucose value to “define” hypoglycemia throughout the entire neonatal period (e.g., up to 4–6 weeks of age). This fails to take into account the normal physiology of glucose regulation over the first days of life and the different mechanisms of hypoglycemia in the pathological conditions found over that same time period. The need to intervene in newborns with low plasma glucose levels must obviously be weighed against possible adverse consequences of disturbing normal feeding behavior and parental-newborn bonding and also has to consider factors such as post-natal age, infant or maternal risk factors, and degree and mechanism of hypoglycemia. In normal newborns, hypoglycemia due to Transitional HI in the first 24–36 h is not accompanied by any compensatory increase in ketones as an alternative fuel. In contrast, in normal breast-fed newborns, during day 2–4 of age [see Figure 1 from (4)], there is a second phase of hyperketonemic hypoglycemia when increased ketones can appropriately compensate for low glucose. Thus, in normal newborn infants, the risk for hypoglycemic brain injury should be evaluated based on several factors: (1) the level of plasma glucose and the level of alternative fuels available such as beta-hydroxybutyrate; (2) the presence of risk factors for persistent hypoglycemia and, (3) the diagnosis of the etiology of the hypoglycemia. For this reason, the glucose thresholds for brain responses to insulin-induced hypoglycemia in adults (2), should be strongly considered as a guide to both the glucose thresholds for intervening (persistently <50 mg/dl [<2.8 mmol/L] in the first 48 h of life and persistently <60 mg/dl [<3.3 mmol/L] after that) and the glucose targets for treatment [>70 mg/dl (>3.9 mmol/L) for those requiring IV glucose to treat severe hypoglycemia]. In addition, the degree of hypoglycemia and its duration are also major factors in choosing when and how to intervene. For instance, for healthy term babies with no risk factors for the development of the persistent forms of hypoglycemia simple methods, such as a feed or oral glucose gel may be sufficient for milder hypoglycemia; whereas for those at risk for persistent hypoglycemia, more invasive treatments, such as IV dextrose bolus and continuous glucose infusions in addition to full oral feeds would be appropriate.

In addition to taking a careful history, monitoring of ketones as well as glucose should be very useful in guiding the need for interventions and appropriate glucose targets during the second phase of hypoglycemia beyond 36–48 h of age, when Transitional HI should normally have resolved. Beyond the fifth day of age, plasma glucose levels should have reached the same normal range as older infants and children, and the same guidelines used at these ages should apply (3).Newborn infants who have hypo-ketonemic hypoglycemia lasting beyond 24–48 h of age should be considered as highly likely to have a persistent hyperinsulinism disorder, either a genetic form of HI or Perinatal Stress-Induced HI.

### 4.2. Improved screening for the persistent hypoglycemia disorders, perinatal stress-induced HI and genetic HI

A sign of major deficiencies in the current protocols for managing neonatal hypoglycemia is the high frequency of permanent brain injury in babies with congenital HI and other persistent forms of hypoglycemia due to delays in diagnosis and treatment. This includes especially infants with Genetic HI, but also babies with Perinatal Stress-Induced HI (10–12, 18, 43). Since the Transitional HI present at birth in all normal newborns appears to resolve between 24 and 36 h of age (Figures 1, 2), it should be feasible to screen for persistent hypoglycemia disorder by combined monitoring of both plasma glucose and ketone levels until either (1) the plasma glucose levels stabilize within the normal range of 70–100 mg/dl [3.9–5.6 mmol/L] OR (2) the plasma levels of beta-hydroxybutyrate (BOHB) are no longer suppressed (e.g., >1–1.5 mmol/L) when glucose is <60 mg/dl [<3.3 mmol/L]. For normal babies with Transitional HI, this appears to occur by 24–36 h (see Figures 1, 2), so the screening procedure need not delay discharge beyond the usual 2 days of age. Infants who fail to demonstrate resolution of hyperinsulinism must not be discharged home, but should remain in the nursery for further monitoring and evaluatoin as this indicates the likelihood of persistent HI (Perinatal Stress Induced HI or Genetic HI), not transitional HI.

### 4.3. Improved strategies for treating neonatal hypoglycemia

Since all of the three most common forms of neonatal hypoglycemia at birth (Transitional HI, Perinatal Stress-Induced HI, Genetic HI) involve hyperinsulinism, interventions for acute management of hypoglycemia immediately after birth should be based on hyperinsulinism as the underlying mechanism. Because hyperinsulinism causes increased glucose consumption, single small doses of glucose (e.g., a feeding or dextrose gel applied to the gums) can be expected to raise plasma glucose concentrations only briefly and are likely either to need to be repeated frequently or to be supplemented with additional measures. The currently recommended treatment for symptomatic hypoglycemia or for asymptomatic hypoglycemia that is very severe or prolonged is intravenous dextrose in addition to continuation of standard oral feeds. There is no evidence to suggest feeding should be discontinued in infants requiring IV dextrose treatment. Rates of glucose utilization in babies with hyperinsulinism often exceed the normal rate of 4–6 mg/kg/min and may be as high as 20–30 mg/kg/min or higher. Therefore, frequent monitoring and adjustment of glucose infusions every 15–30 min may be required.

Glucocorticoids are always ineffective in treating hypoglycemia due to hyperinsulinism (or other forms of hypoglycemia not caused by cortisol deficiency) and should be avoided. Glucagon deserves more detailed exploration as an alternative acute treatment, since hepatic glycogen stores will be retained in hyperinsulinism and glucagon can promptly raise plasma glucose levels for 1–2 h or more. In addition, the use of glucagon should become simpler and more convenient with newer, stable formulations that may be available in the near future (44). Glucagon can be given as mini-boluses, SQ or IV, and newer formulations may be compatible with continuous SQ infusion (lowering the need for placing central IV lines or intragastric tubes for continuous dextrose infusions).

For prolonged hypoglycemia due to hyperinsulinism (>5–10 days), diazoxide is often effective if treatment is needed (18). Careful evaluation of the risks and benefits of diazoxide should be made, particularly in premature infants where the risk of adverse effects may be greater (45–47). Once prolonged hyperinsulinism is detected, consultation with pediatric endocrinology is recommended before discharge to home. As emphasized in the PES guidelines, all infants with a possible hypoglycemia disorder should have a fasting test to prove resolution prior to discharge (3).

## 5. Main points

1. Recognition and treatment of newborns with persistent forms of hypoglycemia are important to reduce the high frequency of permanent hypoglycemia-induced brain damage in these children.
2. All three major forms of neonatal hypoglycemia are caused by hyperinsulinism: Transitional HI in normal newborns, Perinatal Stress-Induced HI, and Genetic HI, and are characterized by low plasma glucose and plasma ketones.
3. A common mechanism involving decreased expression of beta cell K_ATP_ channels explains the lower threshold for glucose-stimulated insulin release in Transitional HI and Perinatal Stress-induced HI, with the two representing two ends of the spectrum of persistent fetal hyperinsulinism, and thus, these two forms of neonatal hypoglycemia cannot be distinguished biochemically on the first day of life.
4. Combined monitoring of both plasma glucose and ketone levels may improve recognition of infants with persistent forms of hypoglycemia prior to discharge from the nursery.
5. Interventions to manage hypoglycemia in neonates should take into consideration the underlying disease mechanism.

## 6. Conclusions

For many years misguided efforts to defining neonatal hypoglycemia as a specific plasma glucose concentration have failed to provide a clear and universally accepted approach to identifying neonates that require intervention to prevent hypoglycemia-induced brain damage. In this review, we provide an alternative approach based on the molecular mechanisms responsible for the three major forms of hypoglycemia in neonates, highlighting the use of betahydroxybutyrate as an additional biomarker to identify neonates at risk for persistent hypoglycemia. We remind the reader to also be aware of the other rare endocrine and metabolic causes of hypoglycemia presenting in the newborn period. We recognize the limitations of the data generated to date presented here and welcome new research to fill these knowledge gaps.
